# Corrigendum to “Molecular heterogeneity in histomorphologic subtypes of lung adeno carcinoma represents a challenge for treatment decision” [Neoplasia 49 (2024) 100955]

**DOI:** 10.1016/j.neo.2026.101363

**Published:** 2026-09-16

**Authors:** Tobias Kolb, Sarah Müller, Peter Möller, Thomas F.E. Barth, Ralf Marienfeld

**Affiliations:** Institute of Pathology, Ulm University, Ulm, Germany

The authors regret not to have added the complete supplementary data.

The authors would like to apologise for any inconvenience caused.

**Figure:**
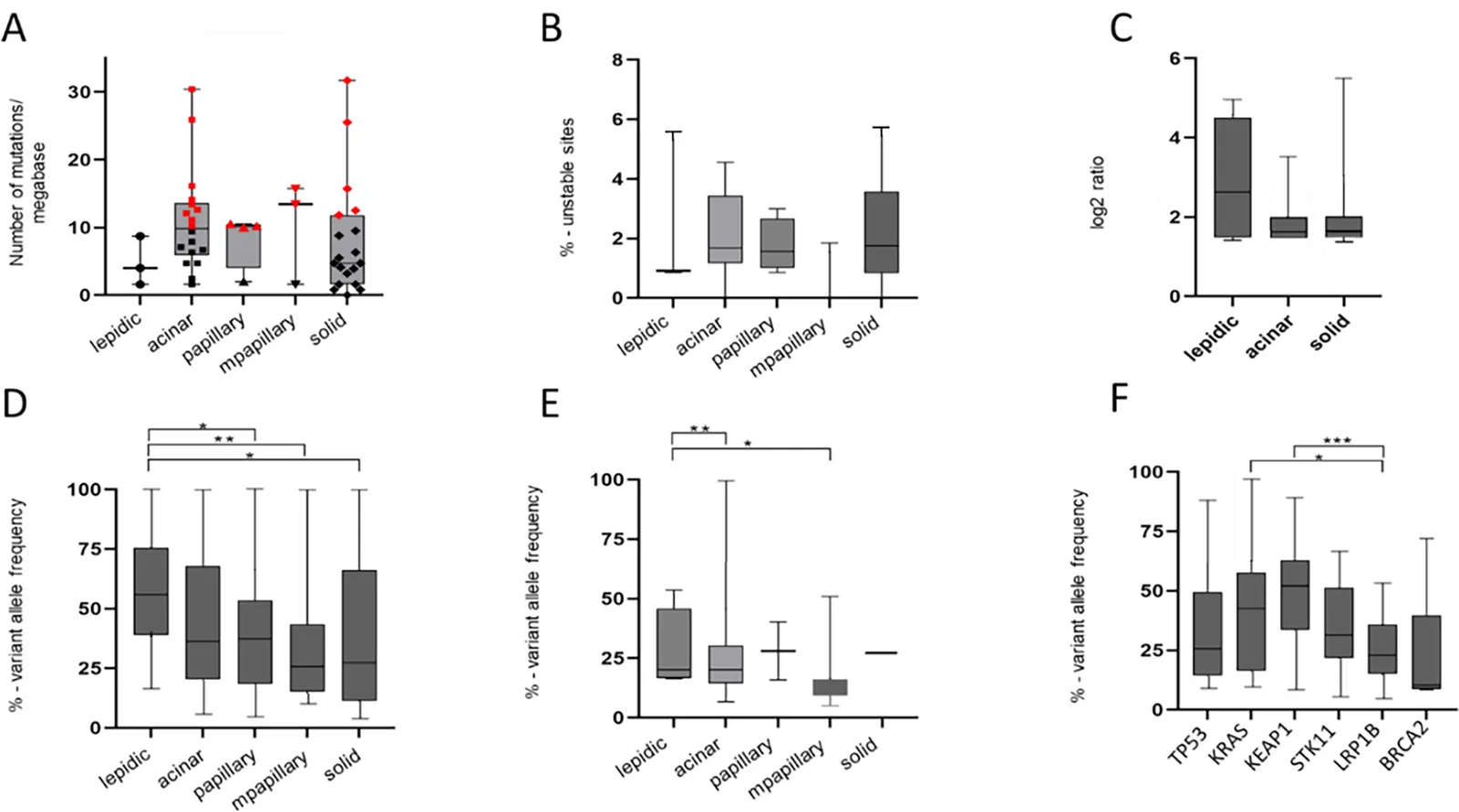

**Supplementary Figure 8: Comparison of diagnostic biomarkers between different growth patterns of NSCLC.** Boxplots of (A) Tumor mutational burden, (B) Microsatellite instability, (C) Copy number variations between the different histologic subtypes, (D) Variant allele frequencies of each mutation classified as uncertain significance, likely pathogen and pathogen, (E) VAF of mutations specific for each growth pattern and (F) VAF of therapy relevant genes are depicted. Data are presented as min–max boxplots, in contrast to the Tukey boxplots used in Figure 2 of the main manuscript. Mean values and standard deviation are shown. Statistical significance: *=p≤0.05, **=p≤0.01.

**Figure:**
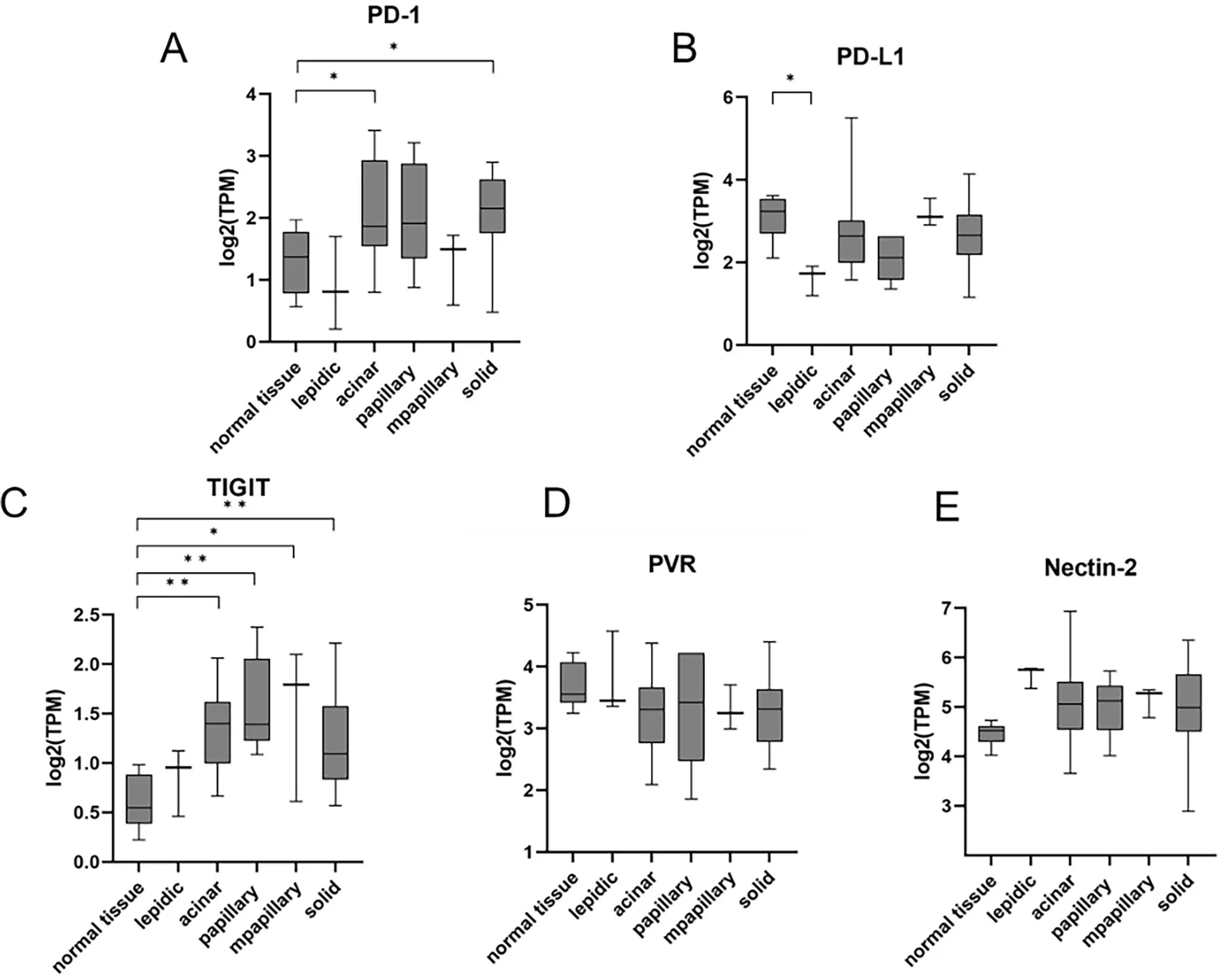

**Supplementary Figure 9: Comparison of immune checkpoint expression between NSCLC histomorphological subgroups determined by RNASeq.** Expression of the different immune checkpoints (A) PD-1, (B) PD-L1, (C) TIGIT, (D) PVR, and (E) Nectin-2 between the different histologic subtypes. Data are presented as min–max boxplots, in contrast to the Tukey boxplots used in Figure 6 of the main manuscript. Mean values and standard deviation are shown. For the min–max boxplots, an additional Dunnett’s multiple comparison test was performed, resulting in some differences in statistical significance compared with Figure 6. Statistical significance: *=p≤0.05, **=p≤0.01.

